# Older adults with constipation and Parkinson’s disease share comparable gut microbiota

**DOI:** 10.3389/fcimb.2026.1688918

**Published:** 2026-07-23

**Authors:** Leyong Ke, Yue Wang, Li Tang, Min Yang, Qinxin Liu, Jianfei Wu, Xiaogang Bi, Yunbo Chen, Xin Liu

**Affiliations:** 1 Department of Cosmetic and Plastic Surgery, Zigong Fourth People’s Hospital, Zigong, China; 2 Department of Gastroenterology, The Affiliated Hospital of Qingdao University, Qingdao, Shandong, China; 3 Department of Gastroenterology, The Affiliated Hospital of Southwest Medical University, Luzhou, China; 4 Department of Neurology, Zigong Fourth People's Hospital, Zigong, China; 5 Zigong Institute of Brain Science, Zigong Affiliated Hospital of Southwest Medical University, Zigong, Sichuan, China; 6 Department of Gastroenterology, Zigong Fourth People's Hospital, Zigong, China

**Keywords:** constipation, GMrepo database, microbiota, older adults, Parkinson’s disease

## Abstract

**Background:**

Aging underlies numerous diseases, including Parkinson’s disease (PD) and constipation. A large proportion of patients with PD often suffer from constipation; however, it remains unclear whether older patients with constipation and those with Parkinson’s disease share common gut microbiota characteristics.

**Aim:**

This study analyzed the characteristics of the gut microbiota in older adults with constipation or PD to identify differences and similarities and to evaluate the possible role of the microbiota in PD and constipation.

**Methods:**

Raw data from fecal samples of 860 selected older adults from the Gut Microbiota Database (GMrepo) were analyzed, including patients with PD (n=378), constipation (CP, n=189), and healthy participants (CON, n=293).

**Results:**

The CP group displayed a higher relative abundance of *Bacteroides* and *Akkermansia*, and a decreased relative abundance of *Prevotella*, *Faecalibacterium*, and *Agathobacterium* than the CON group. Similarly, the PD group exhibited an increased relative abundance of *Bacteroides*, *Akkermansia*, *Parabacteroides*, *Phascolarctobacterium*, and *Bifidobacterium*, and a decreased relative abundance of *Escherichia-Shigella*, *Faecalibacterium*, *Agathobacterium*, and *Prevotella*. Further analysis revealed that, compared with the CON group, most bacteria were enriched or deficient in the CP and PD groups, such as *Bacteroides, Akkermansia*, *Faecalibacterium*, *Agathobacterium*, and *Prevotella*.

**Conclusion:**

The microbiota composition and structure of older adults with constipation or PD were similar and significantly different from those of healthy controls. This may partially explain the coexistence of constipation and PD in terms of the gut microbiota.

## 1 Introduction

Parkinson’s disease (PD) is a rapidly progressive neurodegenerative disorder characterized by motor and non-motor symptoms. Epidemiological data indicate that approximately 200 out of 100,000 individuals worldwide are diagnosed with PD (Titova and Chaudhuri, 2018). By 2050, over 12 million individuals worldwide are anticipated to live with PD (Rocca, 2018), imposing a substantial burden on both individuals and society. The hallmark symptoms of PD are tremors, rigidity, bradykinesia, and postural instability. In contrast to motor symptoms, non-motor symptoms manifest earlier and affect a broader range of systems in patients with PD, primarily presenting as constipation, rapid eye movement sleep behavior disorders, pain, and depression (Leite Silva et al., 2023).

The pathogenesis of PD remains incompletely understood, although it is predominantly attributed to the degeneration and loss of dopaminergic neurons in the substantia nigra of the midbrain. However, this does not fully account for the emergence of non-motor symptoms. The gut-brain axis hypothesis proposed by Braak and colleagues suggests that pathological α-synuclein aggregation may originate in the gastrointestinal tract and subsequently propagate to the central nervous system through the vagus nerve. However, this hypothesis remains controversial, as not all patients with PD exhibit identical pathological progression patterns. Emerging evidence suggests that gut microbiota dysbiosis, intestinal barrier dysfunction, microbial metabolites, and systemic inflammation collectively contribute to PD pathogenesis through bidirectional gut-brain interactions. In particular, short-chain fatty acids may influence neurodegeneration by modulating neuroinflammation, intestinal permeability, and α-synuclein aggregation (Braak et al., 2003; Arnsten et al., 2021). Recent research has further demonstrated significant bidirectional interactions between the gut microbiota and the central nervous system, termed the gut microbiota-brain axis. These interactions critically influence brain function and have profound implications for the development of neurodegenerative diseases (Schneider et al., 2024).

Research indicates that the intestinal microbiota of patients with PD exhibits an overgrowth of *Bifidobacterium*, *Lactobacillus*, and *Akkermansia*, as well as the opportunistic pathogens *Porphyromonas* and *Corynebacterium*. Concurrently, reduced abundances of *Prevotellaceae*, *Lachnospiraceae*, and *Faecalibacterium* have been reported (Nishiwaki et al., 2020). Furthermore, these gut microbiota alterations may contribute to PD pathogenesis via the gut-brain axis (Huang et al., 2023).

Among the nonmotor symptoms of PD, constipation is the most prevalent gastrointestinal manifestation (80%-90%) (Fasano et al., 2015), and represents an early indicator of autonomic dysfunction, preceding the onset of clinical motor symptoms by an average of 15.3 years (Postuma et al., 2013). Moreover, the risk of PD increases with the severity of constipation (Lin et al., 2014). Therefore, targeted management of constipation in older adults may have substantial preventive implications for PD (Yuan et al., 2024).

Constipation is a common clinical disorder characterized by dry, hard stools, difficulty in defecation, reduced frequency of defecation, and excessive straining. A multinational analysis of a large sample questionnaire revealed that the incidence of chronic constipation is positively correlated with age, with a prevalence rate exceeding 20% in individuals aged ≥ 70 years and older (Takeoka et al., 2023). The primary mechanism involves intestinal dysfunction, characterized by inadequate secretion of intestinal fluids, reduced intestinal peristalsis, impaired fluid transport, and dysregulation of enteric nervous system (ENS) conduction (Zhao et al., 2021). Recent research has indicated that gut microbiota dysbiosis plays a significant regulatory role in the pathogenesis of constipation. Initial findings derived from traditional microbial culture methods have demonstrated significant depletion of probiotic genera, such as *Bifidobacterium* and *Lactobacillus*, within the gut microbiota of individuals suffering from constipation. Conversely, abnormal proliferation of conditionally pathogenic bacteria, including Escherichia coli and Staphylococcus aureus, has also been observed (Khalif et al., 2005). Current evidence suggests *Bifidobacterium* and *Lactobacillus* are the predominant microorganisms in the intestinal tract of PD patients with constipation comorbidity (Du et al., 2022). This finding may be associated with the age and body mass index (BMI) of the constipated cohort. Consequently, gut microbiota alterations in individuals with constipation warrant further investigation.

The pathophysiological relationship between constipation, an established prodromal non-motor symptom of PD, and PD development requires further investigation. Additionally, alterations in gut microbiota profiles among constipated older adults may hold utility as biomarkers for early PD diagnosis and therapeutic development, warranting further exploration. To address this, we separately evaluated the characteristics of gut microbiota in patients with PD and constipation. Subsequently, we compared the differences and similarities between the two groups of microbiota, to explore their potential association with PD. Despite increasing evidence linking gut microbiota dysbiosis to both Parkinson’s disease (PD) and constipation, few studies have systematically compared the microbial characteristics shared between older adults with constipation and those with PD using large-scale public microbiome datasets. In particular, it remains unclear whether constipation-associated microbial alterations in older adults resemble those observed in PD, which could provide insights into the prodromal gastrointestinal manifestations of PD and the gut-brain axis. Therefore, in this study, we analyzed gut microbiota profiles from older adults with constipation, PD, and healthy controls using the GMrepo database to identify shared and distinct microbial signatures and to explore their potential relevance to PD pathogenesis.

## 2 Materials and methods

### 2.1 Study participants

GMrepo is a systematically integrated and standardized annotated human gut metagenomic database designed to enhance the reusability and accessibility of extensive metagenomic data (https://gmrepo.humangut.info). Currently, GMrepo has incorporated 58,903 human gut samples from 253 studies, encompassing 92 disease and health phenotypes (Wu et al., 2020).

In this study, we used the GMrepo database to evaluate the characteristics of the gut microbiota in older adults with constipation and PD. Samples from the GMrepo database were included based on the following criteria: phenotypes of “Parkinson,” “constipation,” and “healthy”; participants were older adults aged ≥ 60 years; samples were sequenced using 16S rRNA sequencing (region V4); and the data sheet quality control status was satisfactory. Demographic information, including age, sex, geographic location, and body mass index (BMI), was extracted from the GMrepo database. The final raw data comprised fecal samples from 860 older adults obtained from the GMrepo database.

### 2.2 16S rRNA gene sequencing analysis

Sequence data processing and quality control were conducted using the QIIME2 platform (version 2.0). High-quality reads underwent denoising to remove chimeric sequences, followed by clustering into operational taxonomic units (OTUs) based on a 97% similarity threshold. Taxonomic assignment of the resulting OTUs was performed using QIIME2 with reference to the SILVA database (version 128). Statistical analysis of the processed data was subsequently carried out using the STAMP software (version 2.1.332).

The diversity of gut microbiota in the constipation, Parkinson, and control groups was analyzed, and differences in the composition of gut microbiota among the three groups were examined at the phylum, family, and genus levels. The linear discriminant analysis (LDA) effect size (LEfSe) method was employed to identify key genera, and significant biomarkers distinguishing the samples of the constipation, PD, and control groups were identified using random forest modeling.

### 2.3 Statistical analysis

All statistical analyses were performed using Python. Continuous variables were expressed as mean ± standard deviation and analyzed using Student’s t-test or the Mann–Whitney U test, as appropriate. Categorical variables were expressed as percentages and analyzed using the chi-square test.

## 3 Results

### 3.1 Clinical characteristics

Raw data of fecal samples from 860 participants were used for subsequent analysis, of which 189 and 378 were from the CP and PD groups, respectively. Additionally, 293 healthy individuals were enrolled in the control (CON) group. The average age of the participants was 66.30 years and 46.17% were female. Age was not significantly different among the three groups (*P*>0.05). However, significant differences in gender distribution were observed. The proportion of women was significantly higher in the constipation group than in the CON group (*P* < 0.05), whereas the proportion of men was significantly higher in the PD group than in the CP group (*P* < 0.05). A total of 86.74% of the participants were from the USA, and there were significant differences between the CP and CON groups. The clinical characteristics of the study participants are shown in **Table 1**.

**Table 1:** Clinical characteristics of the participants.

Characteristics	Total	Healthy control	Constipation	Parkinson’s disease
N	860	293	189	378
Age (mean ± SD)	66.30 ± 4.47	66.36 ± 5.09	65.71 ± 4.37	66.49 ± 3.83
Gender				
- Female, n (%)	358 (41.6)	112 (38.2)	117 (61.9)	129 (33.3)
- Male, n (%)	502 (58.37)	181 (61.8)	72 (39.1)	249 (66.4)
Country				
USA	746 (86.74)	293 (100)	75 (39.6)	378 (100)
Not-USA	114 (13.26)	0	114 (60.4)	0

### 3.2 Gut microbial communities in older adults with constipation and PD had similar richness and diversity

**Figure 1:**
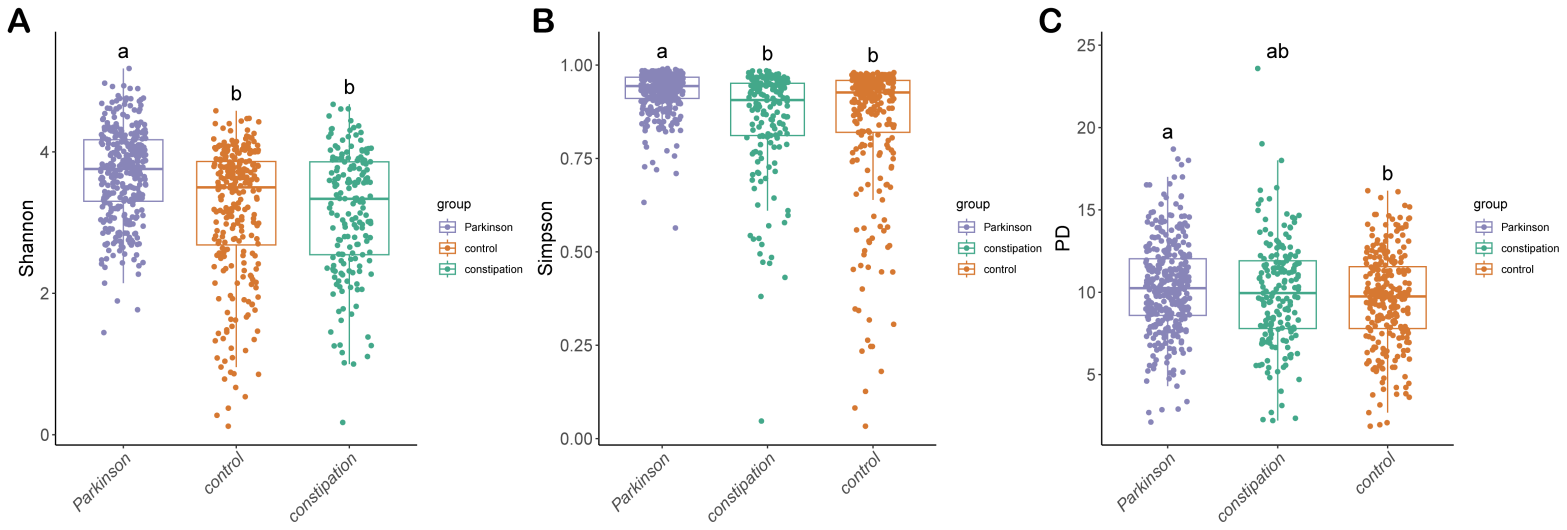
Alpha diversity indices of older adults with Parkinson’s disease, constipation, and healthy control groups. **(A)** Shannon diversity index in the three groups; **(B)** Simpson diversity index in the three groups; **(C)** PD index in the three groups.

Based on the sequencing results, the curves for each sample reached a plateau, indicating adequate sequencing depth. The average community indices of fecal microbiota, including Simpson index (**Figure 1A**), Shannon index (**Figure 1B**), and PD index (**Figure 1C**), were used to assess the α-diversity of each sample group. No significant differences in alpha diversity were observed between the PD and CP groups (p>0.05). In contrast, the PD group demonstrated significantly higher richness and diversity than the CON group (p<0.05). These data indicate that the richness and diversity of the gut microbiota in the CP and PD groups were similar.

### 3.3 Gut microbiota composition across the three groups

**Figure 2:**
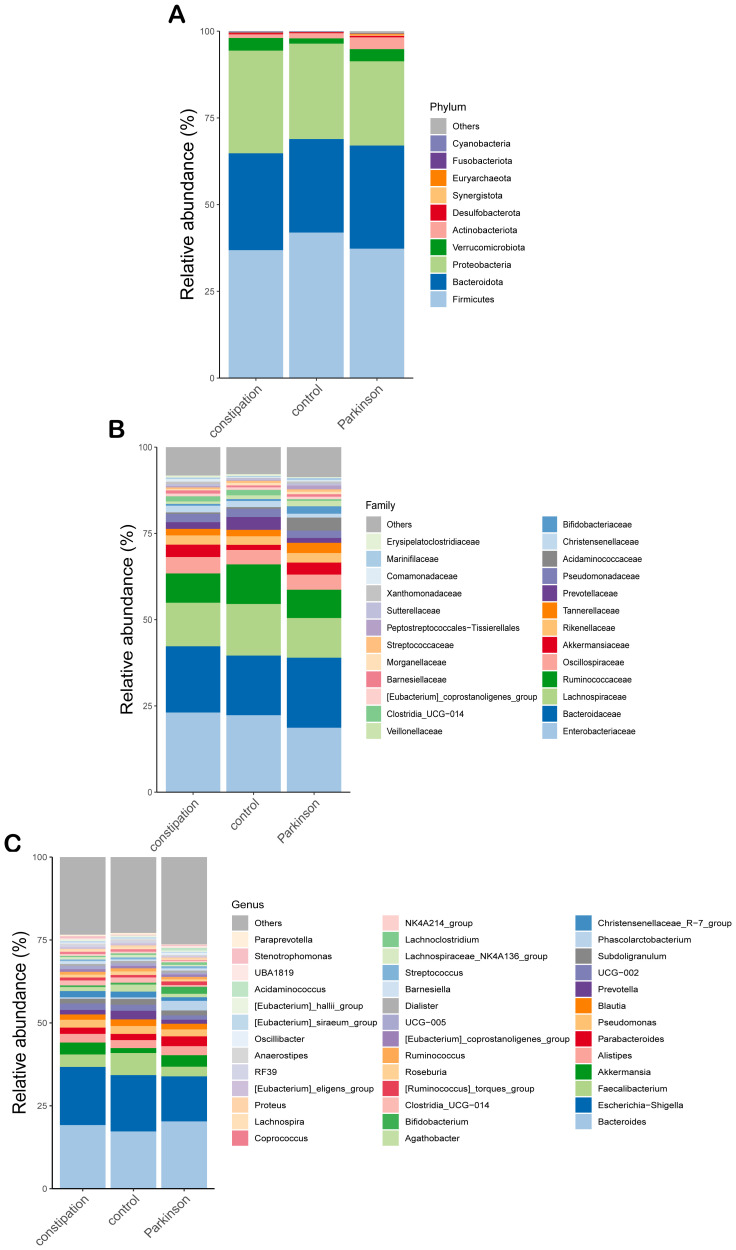
Composition of the intestinal microbiota in individuals with constipation, Parkinson's disease, and healthy controls. **(A)** The ten most prevalent phyla, **(B)** the twenty-five most prevalent families, and **(C)** the 40 most prevalent genera among the constipation, Parkinson’s disease, and healthy control groups.

To further investigate the altered composition of the gut microbiota, taxonomic composition analysis was conducted. Based on the species annotation results, the top ten taxa in each group, in terms of maximum abundance at the phylum level, were selected to generate a bar chart illustrating the relative abundance of species. The dominant phyla in the human intestine were *Firmicutes*, *Bacteroidota*, and *Proteobacteria*, in descending order. Notably, the relative abundance of *Firmicutes* was lower in both the CP and PD groups, whereas that of *Verrucomicrobiota* was higher in the CP group. In contrast, the relative abundances of *Bacteroidota* and *Actinobacteria* were higher in the PD group than in the healthy control group (**Figure 2A**).

Subsequently, the top 25 taxa in terms of maximum abundance at the family level for each group were selected to generate a bar chart of species relative abundance. Compared to the healthy control group, both the constipation and PD groups exhibited an increase in the relative abundance of *Bacteroidaceae* and *Akkermansiaceae*, and a decrease in *that of Prevotellaceae*, *Lachnospiraceae*, and *Ruminococcaceae*. Compared with the constipation group, the PD group showed an increase in the relative abundance of *Tannerellaceae*, *Acidaminococcaceae*, *Bifidobacteriaceae*, *Veillonellaceae*, and *Peptostreptococcales-Tissierellales*, whereas *Enterobacteriaceae*, *Christensenellaceae*, and *Clostridia_UCG_014* were decreased in abundance (**Figure 2B**).

At the genus level, the CP group showed higher abundances of *Bacteroides* and *Akkermansia* and a decreased relative abundance of *Prevotella*, *Faecalibacterium*, and *Agathobacterium* compared to that of the CON group. The PD group exhibited an increased relative abundance of *Bacteroides*, *Akkermansia*, *Parabacteroides*, *Phascolarctobacterium*, and *Bifidobacterium* and a decreased relative abundance of *Escherichia-Shigella*, *Faecalibacterium*, *Agathobacterium*, and *Prevotella* compared with the CON group (**Figure 2C**).

### 3.4 Key genera of microbiota in the three groups

**Figure 3:**
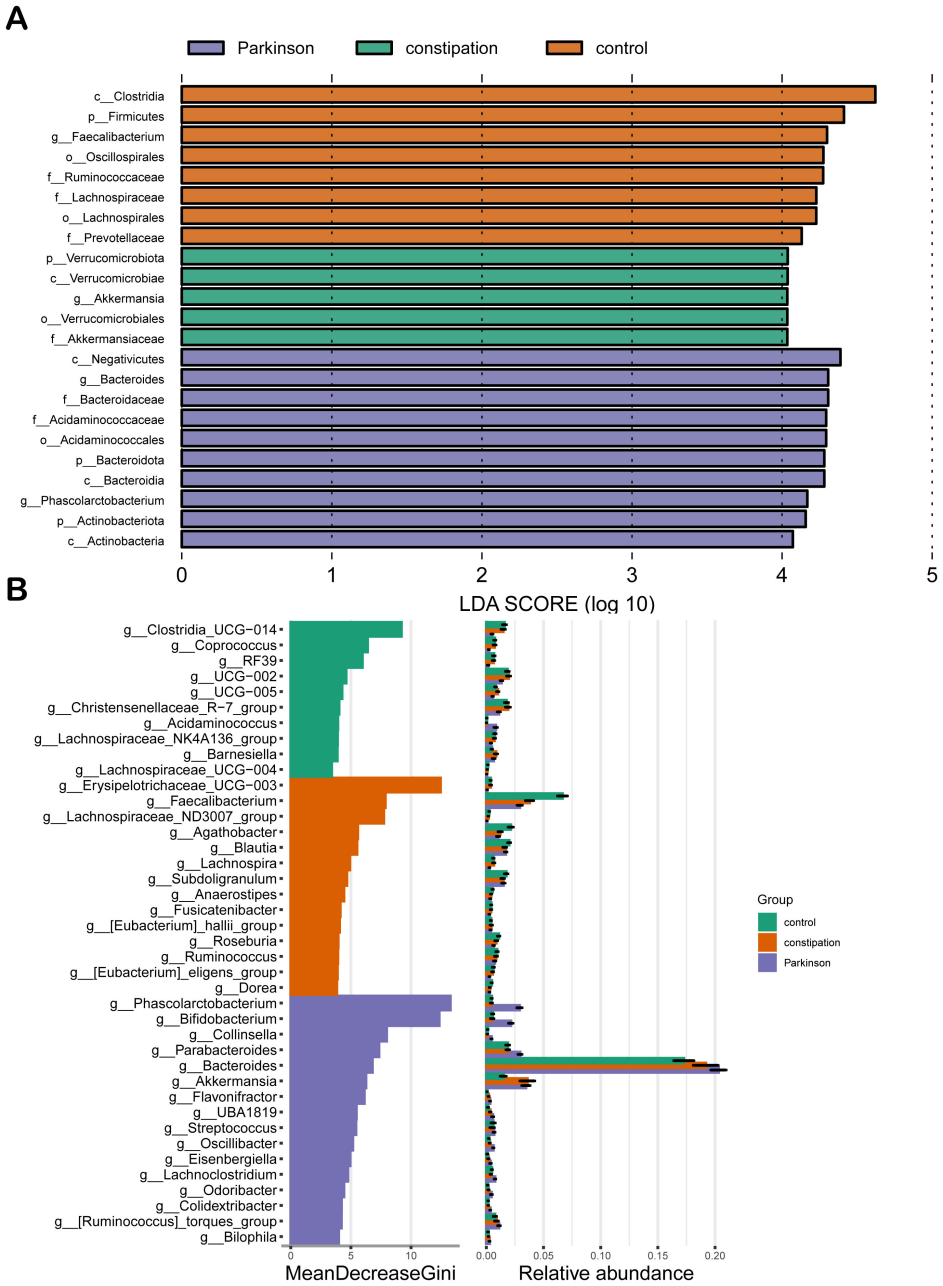
Key genera of microbiota in three groups. The identification of significantly different taxa and the development of a Random Forest (RF) prediction model were undertaken. The mean decrease in Gini index and the relative abundance were employed to assess the relative importance of each genus within the predictive model. **(A)** Differences in bacterial genera in PD, CP, and CON groups. **(B)** Bacterial genera were characterized using mean decrease in Gini index (MDG) and relative abundance analysis.

In this study, we conducted a systematic screening for group-specific differential microbial markers among the PD, constipation, and healthy control groups by employing multi-group comparisons using LEfSe analysis (**Figure 3A**). A hierarchical statistical testing strategy was implemented, initially identifying the overall differential characteristics between groups using the non-parametric Kruskal-Wallis test (α=0.05), followed by pairwise comparisons for validation using the Wilcoxon rank-sum test. To enhance the reliability of the markers, an LDA score threshold of ≥4 was established. It was observed that *Faecalibacterium* was significantly enriched in the healthy control group, *Akkermansia* in the constipation group, and *Bacteroides* and *Phascolarctobacterium* in the PD group (**Figure 3A**).

Subsequently, further analysis was conducted using Random Forest modeling at the genus level, combined with MDG values and relative abundance, to examine the characteristic microbiota of each group (**Figure 3B**). The characteristic microbiota of the constipation group included *Clostridia_UCG_014*, *Coprococcus*, RF39, UCG_002, UCG_005, *Christensenellaceae_R_7*, *Acidaminococcus*, and *Lachnospiraceae*_NK4A136. The healthy controls were characterized by *Erysipelotrichaceae_UCG_003*, *Faecalibacterium, Lachnospiraceae-ND3007, Blautia, Lachnospira*, and *Subdoligranulum*. The characteristic microbiota of the PD group comprised *Phascolarctobacterium, Bifidobacterium, Collinsella, Parabacteroides, Akkermansia, Flavonifractor, UBA1819, Streptococcus, Oscillibacter, Eisenbergiella, Lachnoclostridium, Odoribacter, Colidextribacter*, and *Bilophia*, with the relative and absolute abundances of *Bacteroides* being notably high, thus serving as potential biomarkers for the PD group (**Figure 3B**).

### 3.5 Shared and distinct gut microbiota in PD and constipation

**Figure 4:**
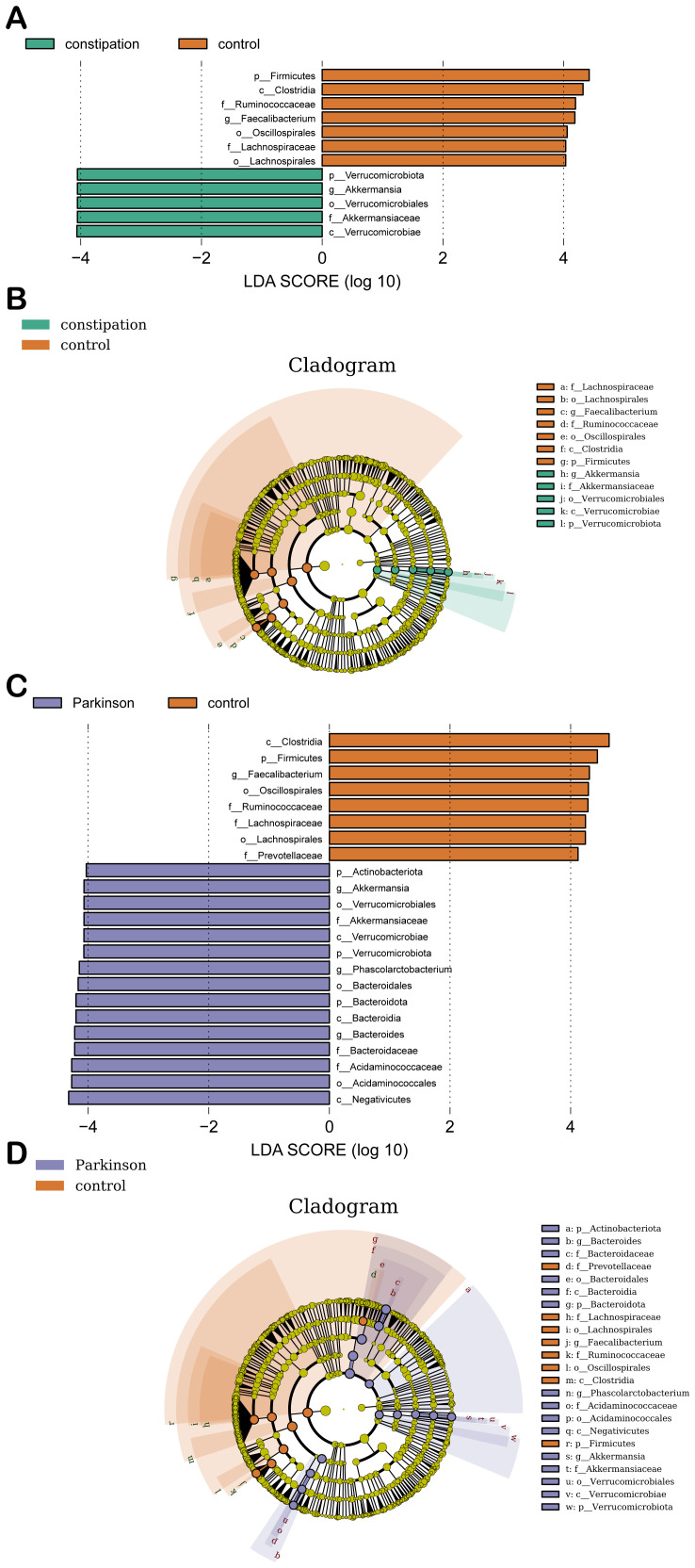
Gut microbiota differences between CP, PD, and CON groups detected by LEfSe analysis. **(A)** A bar graph of linear discriminant analysis (LDA) score distribution showing taxa with LDA scores greater than the set value of 4, and taxa that differed significantly between the CP and CON groups. **(B)** Cladogram showed the differentially abundant species between the CP and CON groups. Concentric circles in the evolutionary branching diagram indicate the taxonomic level from phylum to genus, and the size of each point is proportional to the relative abundance of each taxonomic unit. **(C)** LDA scores showed highly differentially abundant species between the PD and CON groups. **(D)** Cladogram showed the differentially abundant species between the PD and CON groups.

Using LEfSe analysis, we conducted a hierarchical differential comparison of samples from the constipation and PD groups, using the healthy control group as the reference baseline. To mitigate multiple comparison errors and ensure biological significance, an LDA score threshold of ≥ 4 was established, resulting in the generation of LDA histograms (**Figures 4A, B**) and evolutionary branching diagrams (**Figures 4C, D**). Our findings revealed similarities in the gut microbiota between the PD and constipation groups.

**Figure 5:**
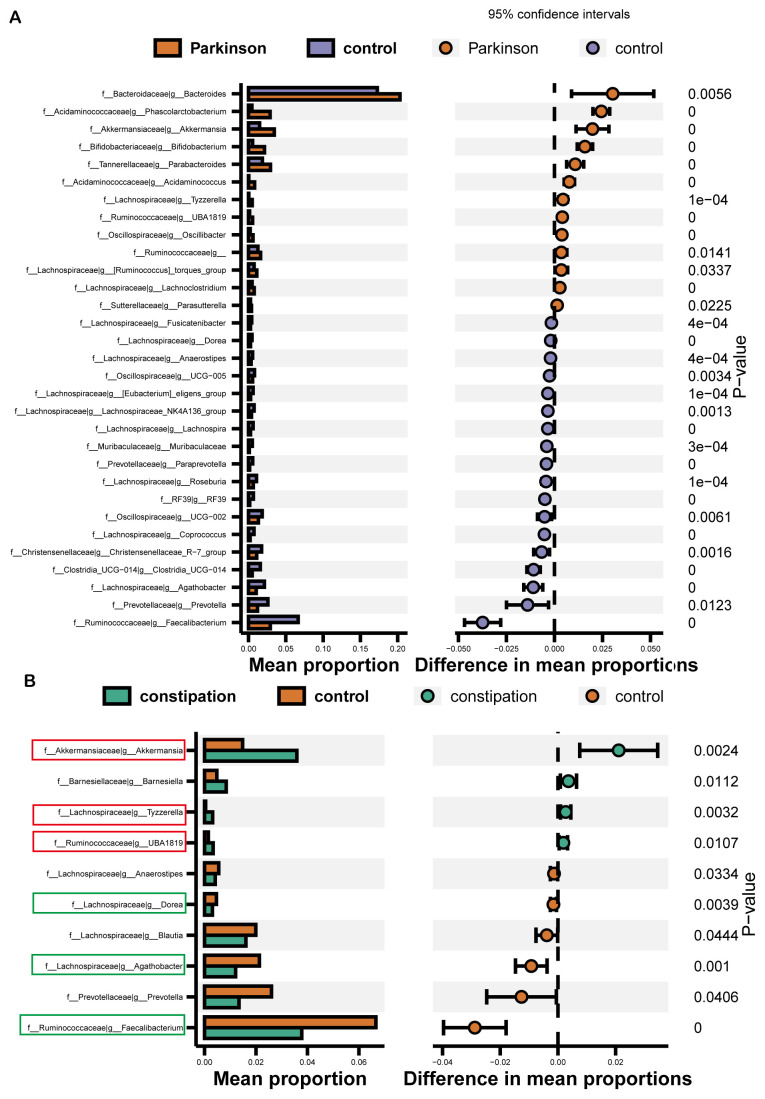
Shared and distinct gut microbiota in PD and constipation. Variations in gut microbiota are observed between the PD group and the CON group **(A)**, as well as between the CP and the CON group **(B)**. The bar on the left side of each graph represents relative abundance. The center of the circle on the right side of each graph denotes the difference in abundance between the two groups. The red boxes indicate that the bacterial species were enriched in the CP and PD groups, compared with the CON group. The green boxes indicate that the bacterial taxa were depleted in the CP and PD groups, compared with the CON group.

At the phylum level, *Actinobacteriota* and *Bacteroidota* were enriched in the PD group compared to the CON group, whereas *Verrucomicrobiota* was enriched in both the CP and PD groups. At the genus level, *Bacteroides* and *Phascolarctobacterium* were enriched in the PD group, and *Akkermansia* was enriched in both the CP and PD groups. Furthermore, this study identified genera with significant differences in both comparisons by analyzing the mean relative abundance of gut microbiota in the PD versus CON groups (**Figure 5A**) and CP versus CON groups (**Figure 5B**). The analysis results indicated that the gut microbiota structure in the PD and CP groups exhibited similar characteristic changes: the genera *Akkermansia*, *Tyzzerella*, and *UBA1819* were significantly enriched in both groups (p<0.05), whereas the genera *Dorea*, *Agathobacter*, *Prevotella*, and *Faecalibacterium* showed a significant decrease (p<0.05) (**Figure 5B**). This suggests that PD and constipation may be characterized by similar gut microecological dysregulation.

## 4 Discussion

This study presents the first systematic comparison of gut microbiota characteristics in older adults with constipation or PD, revealing significant differences in microbial structure and composition. These microbial signatures may serve as potential biomarkers for PD and constipation in older adults. Notably, the gut microbiota profiles of patients with PD and constipation were highly similar, suggesting a shared microbial basis that may contribute to their frequent co-occurrence. Our findings offer a novel microbiological perspective on the potential link between constipation and PD, contributing important evidence to the understanding of the gut-brain axis in neurodegenerative diseases.

Initially, our study identified that patients with PD exhibited significantly higher microbiota richness (ACE index) and diversity (Shannon index) than healthy controls, whereas the constipation group did not show significant differences from controls. This finding contradicts previous studies that reported reduced microbiota diversity in patients with PD. This may be attributed to factors such as the age of the population, disease stage, or the impact of pharmacological interventions (e.g., levodopa). Future studies should validate these findings using clinical medication history and dietary data.

Subsequently, we analyzed the structural composition of the bacterial microbiota at the phylum, family, and genus levels in the three groups. We found that the constipation and Parkinson’s disease groups exhibited significantly increased levels of Akkermansia, Tyzzerella, UBA1819, and Bacteroides compared to the control group, whereas the relative abundances of Dorea, Agathobacter, Prevotella, and Faecalibacterium decreased.

*Akkermansia*, a bacterium of the phylum *Verrucomicrobia*, has emerged as a potentially beneficial microorganism because of its capacity to modulate intestinal immunity and mitigate inflammation by restoring the gut microbiota and maintaining the integrity of the intestinal mucosal barrier (Cani et al., 2022). It has been suggested that *Akkermansia* may ameliorate conditions such as obesity (Dao et al., 2016), diabetes (Plovier et al., 2017), hypertension (Li et al., 2017), colitis, and various age-related complications (Bian et al., 2019). Recent research has indicated that *Akkermansia* is actively involved in several neurological and psychiatric disorders, including depression, anxiety (Sun et al., 2023), multiple sclerosis (Berer et al., 2017), Alzheimer’s disease (Ling et al., 2020) and PD (Fang et al., 2021). Dysregulation of intestinal ecology is observable during the prodromal phase of PD, such as during the onset of constipation, with an enrichment of *Akkermansia* in patients with PD. Additionally, increased levels of *Akkermansia* have been identified in MPTP-induced and fisetinone-treated mouse models of PD (Jeon et al., 2021). These findings suggest that Akkermansia may contribute to PD pathogenesis through its mucin-degrading activity, which can exacerbate intestinal inflammation, impair barrier integrity, and promote endotoxemia and neuroinflammation (Derrien et al., 2017). Thus, the impact of *Akkermansia* on PD may be bidirectional. Furthermore, *Akkermansia* has been found to be relatively abundant in the elderly and positively correlated with aging (Badal et al., 2020). Given that PD and constipation are prevalent among the elderly, it is plausible that increased *Akkermansia* abundance is also associated with aging. Current research on *Akkermansia* and disease is limited to associations, and the existence of a clear causal relationship remains uncertain. Further mechanistic investigations of its role in PD are warranted.

Currently, there are few reports on *Tyzzerella*, and they are controversial. For instance, *Tyzzerella* produces substantial quantities of aromatic amines via the activity of aromatic amino acid decarboxylases (Sugiyama et al., 2022), many of which have the potential to enhance intestinal motility. In a study by Gao et al., *Tyzzerella* demonstrated a positive correlation with GTR and various intestinal barrier factors, while showing a negative correlation with FBST, SS, and several gastrointestinal motility factors (Gao et al., 2023). Conversely, other studies reported a reduced abundance of *Tyzzerella* in constipated mice treated with probiotic formulations containing *Bifidobacterium* or *Lactobacillus*. This was accompanied by an increase in short-chain fatty acid content, a decrease in serum levels of inflammatory factors IL-1, IL-6, and IL-8, and an enhancement in gastrointestinal peristalsis, suggesting a potential association between *Tyzzerella* and the development of constipation (Zhang et al., 2023). Consequently, the role of *Tyzzerella* in constipation and PD, particularly in the elderly population, warrants further investigation. *Ruminococcaceae* have been positively associated with constipation (Zhu et al., 2014). Current research predominantly focuses on obese mice, where UBA_1819 may mitigate inflammation by producing short-chain fatty acids (SCFAs) or other anti-inflammatory metabolites, thereby enhancing intestinal barrier function (Milton-Laskibar et al., 2022). This mechanism may account for its increased prevalence in elderly individuals with constipation and PD.

*Prevotella* is classified under the family Prevotellaceae, whereas Dorea, *Agathobacter*, and *Faecalibacterium* are members of the family *Lachnospiraceae.* These bacterial families are characterized by their ability to produce short-chain fatty acids (SCFAs). SCFAs, primarily butyrate, propionate, and acetate, are the end products of dietary fiber fermentation by the intestinal microbiota and play multifaceted roles in the gut-brain axis. α-Synuclein is a pathological hallmark of PD, and SCFAs, particularly butyrate, have been shown to decelerate the pathological progression of PD by inhibiting aberrant aggregation in the intestinal tract (Duan et al., 2024). Transplantation of gut microbiota from patients with PD exacerbates dyskinesia and neuroinflammation in a transgenic mouse model that overexpresses α-synuclein. Conversely, butyrate supplementation significantly reduces α-synuclein aggregation and ameliorates symptoms (Sampson et al., 2016). Furthermore, SCFAs have the potential to mitigate neurodegeneration by enhancing gut barrier integrity, regulating immune responses, and reducing neuroinflammation (Kalyanaraman et al., 2024). Notably, butyrate has been demonstrated to increase cholinergic neuronal proportions in rats while promoting colonic transport and contraction (Soret et al., 2010), thereby ameliorating constipation.

Although the microbiota composition in individuals with constipation and PD is similar, distinct differences are evident. Notably, the enrichment of Bifidobacterium, Phascolarctobacterium, and Actinobacteriota in patients with PD may be associated with their unique pathophysiological characteristics. For instance, the increase in *Bifidobacterium* is partly attributable to its compensatory role in counteracting the inflammatory environment in patients with PD and may also be influenced by catechol-O-methyltransferase (COMT) inhibitors (Weis et al., 2019). These differences in genera suggest that the microbiota associated with PD is not merely an extension of constipation but may also represent a specific microbial response to neurodegenerative diseases.

In our study, a larger sample size was selected, and the age of the study population was controlled to minimize the effect of age differences on the gut microbiota. However, this study has several limitations. First, this study was based on the GMrepo database, which was unable to assess information such as BMI, dietary intake, food composition, comorbidities, and medication status, whereas obesity, diet, and medication use have important effects on gut microbiota composition (Kwon et al., 2024).

Moreover, the differences in gender and country of the participants may have affected the results of the study. Second, although all samples were sequenced in the 16S rRNA V4 region and passed standardized quality control, residual batch effects across the 253 contributing studies cannot be fully excluded. Furthermore, because the diseases studied here may fluctuate over time, single cross-sectional sampling cannot fully characterize disease-related microbial changes or establish causality. Third, the microbial composition of the various parts of the gut varies slightly, and the microbes extracted from feces do not fully represent the microbial composition of the intestinal mucosa. Finally, 16S rRNA sequencing technology has limited resolution, and in-depth analysis of functional pathways in combination with macrogenomics or metabolomics is required.

## 5 Conclusion

In this study, we conducted a detailed analysis of the gut microbiota in individuals with constipation, PD, and healthy controls. Our findings indicate that the composition and structure of the gut microbiota in older adults with constipation and those with PD exhibit significant similarities. These shared characteristics include significantly increased abundance of *Akkermansia*, *Tyzzerella*, and *UBA1819*, whereas *Dorea*, *Agathobacter*, *Prevotella*, and *Faecalibacterium* exhibited reduced abundance in both groups. Future research should aim to further elucidate the roles of gut microorganisms in the pathogenesis of constipation and PD in older adults. Additionally, it is important to explore the characteristics of dynamic changes in the microbiota during the progression from constipation to PD in elderly patients and to establish microbiota-based markers for PD.

## Data Availability

The datasets analyzed in this study are publicly available in the GMrepo database (https://gmrepo.humangut.info). The data used in this study were obtained from publicly available datasets within GMrepo and can be accessed through the database website and the original studies referenced therein.
